# Analysis of Mercury Concentration in Cosmetic Clays

**DOI:** 10.3390/toxics13060507

**Published:** 2025-06-16

**Authors:** Agnieszka Fischer, Barbara Brodziak-Dopierała, Wiktoria Jańska, Luiza Jeyranyan, Beata Malara

**Affiliations:** 1 Department of Toxicology, Toxicological Analysis and Bioanalysis, Faculty of Pharmaceutical Science, Medical University of Silesia, 30 Ostrogórska Str., 41-200 Sosnowiec, Poland; bbrodziak@sum.edu.pl (B.B.-D.); s76946@365.sum.edu.pl (W.J.); s81890@365.sum.edu.pl (L.J.); 2 Department of Cosmetology, Faculty of Medicine, Wojciech Korfanty Upper Silesian University, Harcerzy Września 1939 no. 3 Str., 40-659 Katowice, Poland; beata.malara@akademiagornoslaska.pl

**Keywords:** cosmetic clay, mercury, cosmetic safety, human exposure

## Abstract

(1) Background: Clays are popular raw materials of natural origin used in cosmetology, beauty salons, and home care. They have moisturizing, soothing, cleansing, disinfecting, detoxifying, and regenerating properties, and can be used externally in the form of poultices or internally in solution form. Though they are characterized by a rich and diverse mineral composition and are considered safe for the body, their use can expose users to harmful elements including mercury. (2) Materials and methods: This study analyzed mercury (Hg) concentrations in samples of cosmetic clays available on the Polish market. Hg analysis was performed using the AAS method with an AMA 254 analyzer. The clays differed in type/color and were purchased from different manufacturers. (3) Results: The mean Hg content in all the tested samples was 28.91 µg/kg, with a range of changes of 1.87–200.81 µg/kg. The highest concentrations of Hg were found in green (AM = 53.26 µg/kg) and white (AM = 52.80 µg/kg) clays, while the lowest were detected in purple (AM = 2.56 µg/kg) and blue (AM = 3.69 µg/kg) clays. The differences in Hg content between individual types of clay were statistically significant. (4) Conclusions: Due to the presence of Hg found in all the samples of cosmetic clay tested, it is likely that these products need to be tested for their metal contents.

## 1. Introduction

Natural resources have long been reliable providers of various raw materials used in medicine and cosmetics. Clay minerals obtained from the earth’s crust were used even in ancient times, where they were applied for facial and body care [1,2]. Gradually, they found their application [3,4] in natural medicine, which widely describes the positive effects of minerals on the human body [5,6].

Clays are created through the hydrothermal transformation of rocks and volcanic ash. They are mainly composed of silicates and aluminosilicates. They may contain admixtures of other minerals, such as quartz, mica, opal, volcanic ash, zeolites, fossils, mud, and sand [4,7,8,9,10,11]. Clays used in medicine and cosmetics are extracted from depths of 20 to 70 m in various geographical locations, with both parameters influencing their mineral composition [2,12]. They are a valued raw material of natural origin considered to be safe for the body [5]. In order to prepare a commercial product, clays are subjected to roasting, grinding, and drying while undesirable substances and bacterial spores are removed [4,7,8,13]. The finished product takes the form of a dusty powder [14].

Clays can be used externally, in the form of masks, compresses, or poultices, as well as internally in the form of solutions. In cosmetic products, they are used as main, active, and additional ingredients, influencing the color or stability of the product, for example. Further, they are used in the formulation of various cosmetic products, such as emulsions, gels, pastes, ointments, powders, and lotions, as well as in shampoos, deodorants, creams, masks, soaps, and toothpastes [1,15]. Clays do not cause skin irritation or dryness and are safe for those who suffer with allergies [4,7]. Moreover, they have a cleansing effect on the skin, absorb impurities, regulate sebum secretion, reduce the risk of blocking sebaceous glands and acne [1,16,17,18], and can be used as a component of sunscreen [16,17]. While, on the one hand, used to moisturize, nourish, and regenerate the skin [15,18,19,20], clays can also be used as wound healing aids [19], as a blood clotting agent and antihemorrhagic agent [21]. Internally, they are used in the treatment of inflammation and diarrhea [22], as well as in detoxification processes [23].

Cosmetic clays take on different colors related to their mineral composition [15,24]. The simplest and most commonly used clay classification method concerns color [25], which is not only an external factor used to differentiate clays, but is also used to determine the product’s effect on the skin [15,26]. Clays’ natural origin determines their contents, and high adsorption capacity may be associated with the risk of harmful compositional elements, including mercury (Hg) [15,27].

The use of Hg in cosmetics is permitted, with limitations. Such regulations vary by country: in China, Korea, the Southeast Asian Nations, and USA, the limit is 1 ppm; in Thailand, it is 0.5 ppm; India limits such use to 0 ppm; and Canada allows for use up to 3 ppm. Japanese regulations do not currently stipulate trace materials in final products [28,29].

Environmental Hg contamination is one of the most worrisome environmental problems currently being faced [30,31]. Studies conducted by Oloruntoba et al. (2024) [32] demonstrate that the southern US is one of five areas of the greatest conservation concern for Hg stress. In this region, the average mono-methyl Hg concentration in fish tissues exceeded limits considered safe for human consumption.

Exposure to mercury, a toxic element, may lead to damage and dysfunction in various body structures, and multifaceted health consequences [33]. Hg toxicity is associated with the disruption of redox homeostasis and microtube assembly. In addition, it may affect intracellular calcium homeostasis, the cytoskeleton, mitochondrial function, oxidative stress, neurotransmitter release, and DNA methylation [33]. Hg exposure results in neurological damage in the cerebral cortex and cerebellum, leading to dysfunction of the central nervous dysfunction. Such changes may also affect other organs, such as the kidneys or the cardiovascular system [34,35,36,37,38,39,40].

The aim of this study was to determine the Hg content in commercially available cosmetic clays. In addition to their recognized health and cosmetic effects, features like low price and high market availability make these products popular both in specialist salons (cosmetology, biological regeneration) and in home care [7,9]. The use of products contaminated with Hg leads to bodily exposure and possible adverse health consequences. Herein, we aim to provide data on the contamination of cosmetic clays with Hg and the importance of these products as potential sources of exposure to this element.

## 2. Materials and Methods

### 2.1. Materials

The tested material consisted of samples of cosmetic clays taken from the random products available on the Polish market. In total, 111 different commercial products were used for the study, differing in color and manufacturer. The selection criteria included easy buyer accessibility, a wide range of type/color offered by the producer, and offer repeatability in other producers. Individual products for testing were selected randomly. A limitation of this study was the inability (a) to purchase a complete set of clay types from each manufacturer and (b) to assess inter-batch variability. The list of tested products and their manufacturers is presented in Table 1.

**Figure 1:**
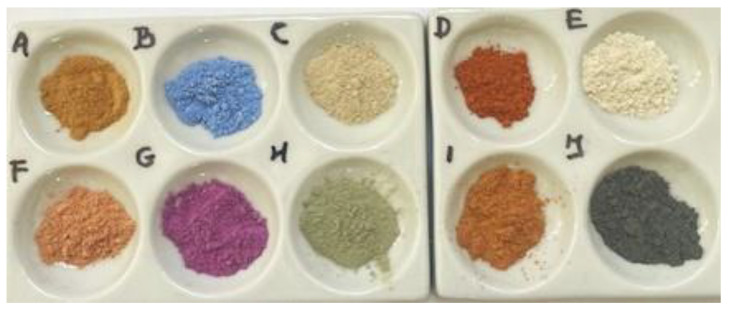
Test samples of different types of cosmetic clays: A—yellow, B—blue, C—Rhassoul, D—red, E—white, F—rose, G—purple, H—green, I—orange and J—black.

We analyzed 10 different types of cosmetic clays. The differentiating factor was color. Red, green, purple, blue, pink, white, black, yellow, and orange clays were tested. Rhassoul (Ghasoul) clay was also tested among the samples, as shown in Figure 1.

Green (19%), red (16%), and white (14%) clays were the most numerous; purple and yellow clays accounted for 11% of all the tested samples. The smallest share was held by orange (3%), Rhassoul, and black (5%) clays. The tested samples came from 9 different manufacturers: Yasumi, Olvita, Nacomi, NaturPlanet, MyBuddy, EcoFlores, EcoSpa, Your Natural Side, and Nature Queen. Most types of clay came from EcoFlores (N = 27), Your Natural Side (N = 24), and Nature Queen (N = 27), while the least came from Olvita, NaturPlanet, and MyBuddy (N = 3), as shown in Table 1.

### 2.2. Methods

The Hg content of clay samples was determined by way of atomic absorption spectrometry (AAS) using the AMA 254 analyser (Altec, Praha, Czech Republic). During the analysis, the total amount of Hg in the sample was determined, though it was not possible to differentiate the forms of Hg occurrence (organic/nonorganic). The instrument used an automatic internal calibration system [41] to determine the Hg concentration in the samples tested, with the lower limit of detection (LOD) being 0.01 ng Hg [41,42].

Before each measurement, in accordance with the analytical procedure, the instrument was cleaned with air and pure water (Elix Essential Water Purification, Merck KGaA, Darmstadt, Germany).

Due to the fact that the clays were sold in a loose form (ground, homogeneous powder), the tested material did not require additional processing before analysis. Three random test samples were prepared from each purchased product.

Approximately 50 mg was weighed for analysis (analytical balance, OHAUS, Parsippany, NJ, USA), with the following measurement conditions used: wavelength: 253.65 nm; carrier gas: oxygen (O_2_ purity ≥ 99.5%); inlet pressure: 200–250 kPa. The individual analysis stages were counted in seconds [s]: drying—200 [s]; decomposition—250 [s]; measurement—90 [s]. The certified reference material INCT-MPH-2 Mixed Polish Herbs (Institute of Nuclear Chemistry and Technology, Warsaw, Poland) [43] was used to validate the assays. The results from six repetitions of the assays were as follows: 0.018 ± 0.002 mg/kg; recovery 92.22%.

### 2.3. Statistical Analysis

The amount of Hg determined in all tested samples was in the range of 0.09–10.08 ng. Hg concentration was determined based on the mass of individual samples (50.06–50.86 mg). Each sample was tested three times. The final Hg concentration in each sample was the arithmetic mean of 3 measurement results. Statistical analysis was performed on 111 samples using Microsoft Excel and Statistica ver. 13.3 pl (Statsoft, Cracow, Poland) (TIBCO Software Inc., Palo Alto, CA, USA, (2017); Statistica, data analysis software system, version 13.3.0) [44].

The distribution of variables was evaluated by using the Shapiro–Wilk test and a quantile–quantile plot. To compare statistical variability, the nonparametric Mann–Whitney U test (for two samples) and Kruskal–Wallis test (for a greater number of samples) were used. Statistical significance was set at *p* < 0.05 [45]. The interval data were expressed as the median (Me), lower (Q_1_), and upper quartiles (Q_3_). The minimum (Min) and maximum (Max) values, the arithmetic mean (AM), and the coefficient of variation (CV) were calculated. The values are accurate to 2 decimal places.

## 3. Results

The Hg content of all the samples tested was in the range of 1.87–200.81 µg/kg, AM (arithmetic mean) = 28.91 µg/kg and Me (median) = 23.72 µg/kg. The CV (coefficient of variation), equal to 136%, indicates a large variability of Hg content in the samples tested. The descriptive results of the statistical analysis of Hg content in all the samples of tested cosmetic clays and divided into clay types are presented in Table 2, divided by clay type. The highest average Hg content was in the green (AM = 53.26 µg/kg) and white clays (AM = 52.80 µg/kg). The median contents for green clay (32.24 µg/kg) and white clay (5.31 µg/kg), which differ from AM, indicate a large differentiation of this feature. In these types of clays, the degree of Hg content differentiation was the greatest, at CV = 108% and 123%, respectively. The N (number of samples) of green and white clays tested was 21 and 15, respectively. For red clay samples, the number in relation to the total number of samples (N = 111) was the largest (N = 18): CV = 52%, AM = 19.71 µg/kg, Me = 21.29 µg/kg. Among the types of clays tested, the lowest average Hg content was recorded in blue clay (AM = 3.69 µg/kg) and purple clay (AM = 2.56 µg/kg). The differences between the Hg content in the individual types of clays tested were statistically significant (Kruskal–Wallis test, *p* < 0.05).

**Figure 2:**
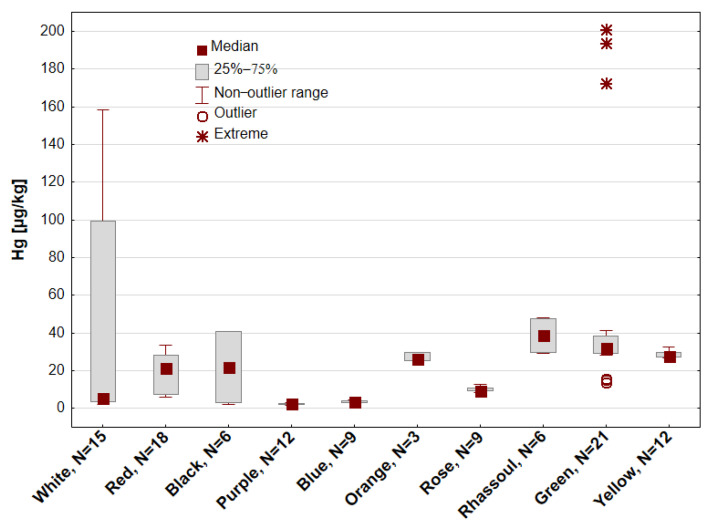
Hg concentration [µg/kg] in cosmetic clays with regard to clay type. N—number of samples.

Figure 2 presents a comparison of Hg concentrations in the samples of tested cosmetic clays with regard to color.

The differences in Hg concentration were statistically significant at *p* < 0.001. Differences were found between the clays as follows: purple and white (*p* < 0.05); purple and red, rose, green, yellow (*p* < 0.001); blue and green (*p* < 0.001); blue and Rhassoul (*p* < 0.001). In Rhassoul clay, the median Hg was the highest (38.72 µg/kg). A high concentration of Hg, exceeding 20 µg/kg, was also recorded for the samples of green, orange, yellow, black and red clays. In purple and blue clays, the average Hg content was the lowest and did not exceed 4 µg/kg, and that in rose clay was higher (Me = 9.56 µg/kg). For the tested samples of white clay (Me = 5.31 µg/kg), the Q_1_–Q_3_ (lower and upper quartiles) (3.51–99.39 µg/kg) and non-outlier values (2.31–158.42 µg/kg) were very large—the largest among all types of samples. In green clay samples, extreme values exceeding 170 µg/kg (exactly, 172.34 µg/kg, 193.72 µg/kg, and 200.13 µg/kg) were recorded, as well as outliers that were lower than the median, with a Hg content of about 14 µg/kg.

**Figure 3:**
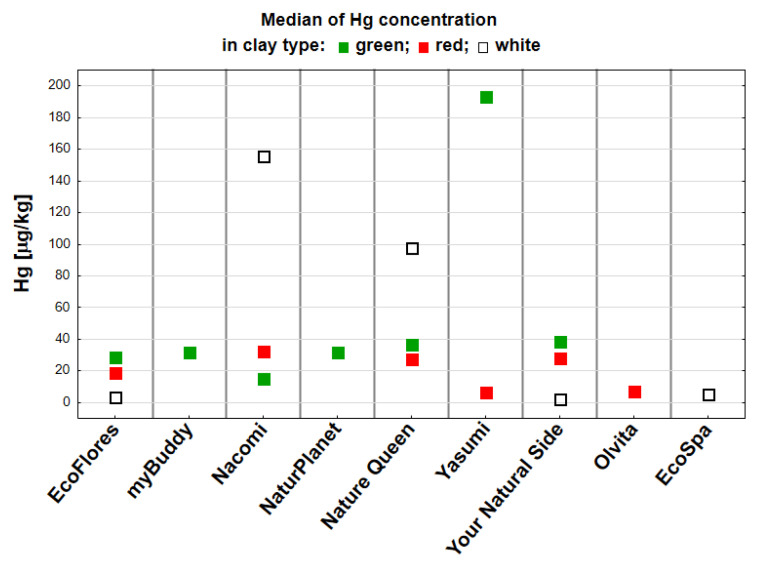
A comparison of Hg concentrations [µg/kg] in white, red, and green clays by manufacturer (in alphabetical order). The results are presented as medians, and statistical significance refers to the manufacturers of a given type.

The analysis of the global cosmetic clay market indicates that the most popular types are white, green, and red [46]. Figure 3 shows the concentration of Hg in the tested samples of these clays from different manufacturers. In these samples, the changes in Hg content depending on the manufacturer were statistically significant. For white clay, the highest Hg concentration was measured in samples from the manufacturer Nacomi (155.90 µg/kg), followed by those from Nature Queen (97.73 µg/kg). In white clay samples from other manufacturers, i.e., EcoFlores, EcoSpa, and Your Natural Side, the Hg concentration was many times lower, in the range of 2.52–5.31 µg/kg. Statistically significant differences were detected for white clay from Yasumi and Your Natural Side (*p* < 0.05). In the case of green clay, the Hg concentration of Yasumi samples (193.65 µg/kg) was statistically higher than in samples from Nacomi (15.06 µg/kg) (*p* < 0.01). The red clay samples tested were characterized by less quantitatively diverse Hg levels. Differences in Hg concentration from 7.26 µg/kg (Yasumi) to 32.29 µg/kg (Nacomi) were statistically significant (*p* < 0.05).

**Figure 4:**
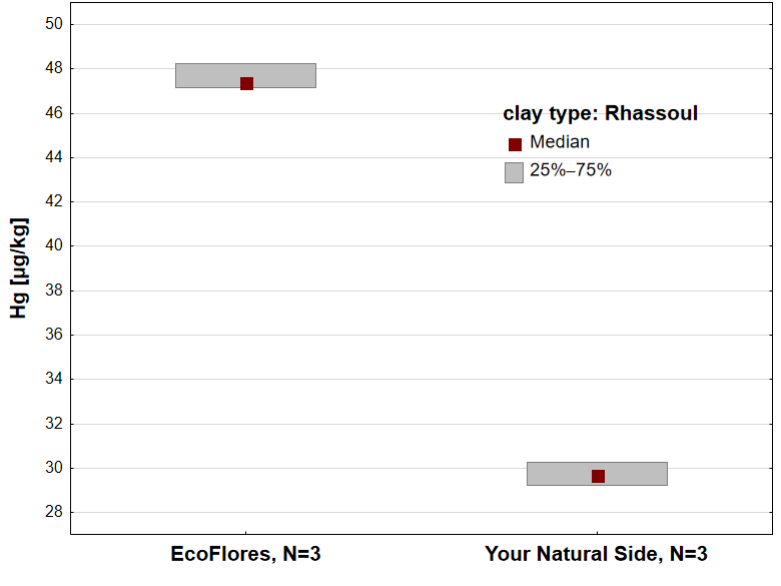
Hg concentration [µg/kg] in Rhassoul clays from EcoFlores and Your Natural Side; N—number of samples.

Figure 4 shows the Hg concentration in Rhassoul clay. This clay is obtained from a strictly defined geographical area in Morocco, and is particularly valued in cosmetology due to its excellent absorption properties [2,12], being used in many cosmetic products, mainly those with cleansing properties [47]. Across the analyzed Rhassoul clay samples, Hg concentration (Me = 38.72 µg/kg) was higher than in the other types of clays tested. This study analyzed Rhassoul samples from two manufacturers. The differences in Hg concentration from EcoFlores (Me = 47.38 µg/kg) and Your Natural Side (Me = 29.67 µg/kg) samples were on the border of statistical significance (*p* = 0.05).

**Figure 5:**
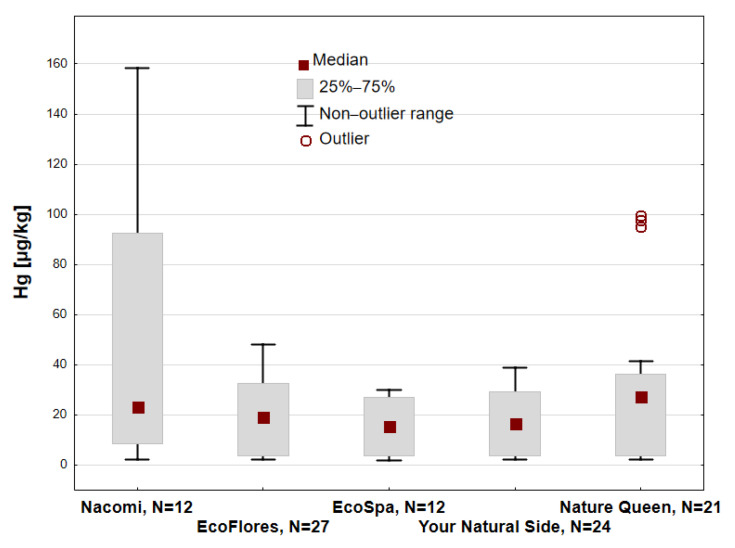
Hg concentration [µg/kg] in different types of cosmetic clay by manufacturer; N—number of samples.

Due to different commercial offers, it was not possible to obtain the same types of clays from all manufacturers. The largest number of clay samples tested came from EcoFlores, and then, in descending order, from Your Natural Side, Nature Queen, EcoSpa, and Nacomi (Table 1). Figure 5 shows the Hg concentrations of all samples tested from these five manufacturers.

The median was the highest from Nature Queen samples (Me = 27.44 µg/kg), and similar values were obtained from Nacomi, Your Natural Side, and EcoSpa samples (15.59 µg/kg). Additionally, outlier values of 100 µg/kg were recorded from samples from Nature Queen. It is worth noting that the largest range of Hg concentration in clay samples was from Nacomi in the Q_1_–Q_3_ range 58.43–92.51 µg/kg.

## 4. Discussion

Currently, there is great interest in natural products across various sectors. Increasing consumer awareness of such products, including in cosmetics [25,47,48,49], leads them to choose natural ingredients more often. The constant search for beauty and youthfulness means that cosmetology is developing dynamically, and the number and variety of products in this field is gradually increasing [50]. The cosmetics market has seen a growing popularity of home care and cosmetic treatments, especially since the COVID-19 pandemic. Meanwhile, the popularity of DIY care and spa treatments using attractive natural products is considered a key factor in the growth of the cosmetic clay market [25].

Cosmetic clays are raw materials of natural origin with varying mineral compositions [2,12], but research results mainly tend to provide information on their nutrient contents. Due to their high adsorption capacity, clays can accumulate toxic substances, such as heavy metals like Hg, meaning natural clay deposits used for cosmetics are rarely pure and may vary in chemical composition [15,51,52]. The problem of harmful pollutants being present in cosmetic clays has not been sufficiently managed.

In our study, Hg content was detected in every sample, with a mean concentration of 28.91 mg/kg and a range of 0.002–0.201 mg/kg. Hg is absorbed into by body via all available routes (alimentary, through inhalation, and through the skin), is cumulative, and induces harmful effects [32,34,37,38,39,40,53]. The Food and Drug Administration (FDA) allows the use of Hg compounds as preservatives only in a few products intended for use on the eye area. In other products, trace Hg contents, not exceeding 1 ppm (0.0001%), can occur as a result of manufacturing [54]. European Union regulations allow contents of 0.007% Hg in the form of preservatives, and from the contamination of ingredients, natural or synthetic, the manufacturing process, storage, and migration from packaging, which is unavoidable for technological reasons [55]. In our study, none of the samples tested exceeded the permissible standards.

Numerous studies indicate the presence of Hg and other heavy metals in cosmetics intended for both care and make-up. Gyamfi et al. (2023) [28] analyzed works on the content of heavy metals in cosmetics. In terms of Hg content, the obtained results varied from being undetectable to a maximum of 90.32 mg/kg in hair dyes [28,56]. High mercury values were also found in products such as lipsticks (30.00–80.00 mg/kg), face powders (48.99–60.77 mg/kg), and eye liners (42.63–67.42 mg/kg) [28]. Similarly, in the study by Parmar and Patel (2024) [57], particularly high levels of Hg were found in eyebrow pencils and eye liners (max. 67.42 mg/kg), and even higher in eye shadow (max. 181.0 mg/kg). Lower Hg concentrations were detected in facial powder in a study in Ghana (mean concentration 2.82 mg/kg) [58], in foundation in Qatar (the range of 0.005–0.0053 mg/kg) [59], and in foundation (0.49 mg/kg), face powder (1.21 mg/kg), and lipstick (5.42 mg/kg) in Pakistan [60]. Cosmetic products, mainly those used for make-up, may contain natural ingredients and clays in addition to other ingredients [57]. In beauty creams, the Hg concentration varied in the range of 47.17–124.8 mg/kg [57]. Hg concentrations were detected in 16 of 19 creams tested by Mohammed et al. (2024) [61] (from 0.294 to 14,414.5 mg/kg) and was not detected in only 3 products. The risk assessment conducted in this study showed that three samples were not safe to use. Furthermore, this study showed that many creams contained Hg, even if it was not listed in the product composition [61]. In our study, there were differences in Hg concentration in cosmetic clays from various manufacturers. Technological process, product preparation, and storage may vary between producers. Contamination with Hg may occur as a result of improper purification of natural raw materials, which are components of cosmetics, and during the production of cosmetic products. Despite the observance of production principles, under which numerous variables are controlled, Hg still appears in cosmetics [62], allowing it to be absorbed into the body. In people using creams containing Hg, an increase in the metal’s content in blood and urine has been observed [63].

In our study on cosmetic clays, a high variation in Hg content was also found (0.002–0.201 mg/kg). The average amounts of Hg for all samples were lower than in the case of the cited studies on cosmetics [60,61,64]. Unlike creams or colored cosmetics, the cosmetic clays investigated in this study are products of natural origin only, though it can be assumed that they have environmental element contents. Moreover, the products analyzed in the study were pure, i.e., single-component products, without other ingredients. The technological processes used in the preparation of multi-component cosmetics are more complex than those in the production of commercial clays [4,7,8,13,15,35,65,66,67], which may affect the content of undesirable substances.

Podgórska et al. (2021) [64] showed that median Hg concentration was higher for natural cosmetics than for conventional cosmetics but this was not statistically significant. As indicated by Mohhamed et al. (2024) [61], in the creams the authors examined, manufacturers sometimes deliberately add Hg compounds without listing these components on product labels. In cosmetics based on natural ingredients, contamination may result from their environmental origin, as indicated by marked contents of heavy metals like cadmium, lead, nickel, arsenic, and mercury in various raw materials [68,69]. Among the cosmetics examined by Ahmed M. et al. (2024) [70] were natural products, such as mud masks, in which the Hg content ranged from 0.022 to 0.061 mg/kg, with a mean of 0.031 mg/kg. These contents were comparable to those examined in cosmetic clay samples in the present study (AM = 0.029 mg/kg). An in-depth analysis conducted by Ahmed et al. (2024) [70] on the metal and metalloid contents in cosmetics emphasizes the need to limit excessive use of cosmetics, the necessity of following proper production practices, the importance of monitoring and regulating metal content to ensure consumer safety, and the impact they have on the environment.

In one cosmetics study, Podgórska et al. (2021) [64] assessed chronic exposure to Hg assessed by calculating chronic daily intake (CDI) and hazard quotient (HQ). The CDI for body cosmetics was 8.72 × 10^−12^, and for face cosmetics, it was 9.56 × 10^−15^, while the HQ was 6.69 × 10^−9^ and 7.36 × 10^−12^ for body and face preparations, respectively. These values were below the acceptable safety standards for cosmetics. On the other hand, the authors suggested that as Hg is a toxic element, exposure to even a small amount constitutes a human health hazard [64]. In our study, although none of the cosmetic clay samples tested exceeded regulatory limits, their potential risks cannot be ruled out. It is generally accepted that Hg is absorbed from cosmetic products locally through intact skin; although a single occurrence of skin contact with a cosmetic product containing Hg compounds is usually not associated with more serious side effects, the long-term use of these products may lead to their accumulation, causing various health problems [64], especially when clays are simultaneously used internally.

In the case of cosmetics used around the lips, for example, the product may be partially swallowed. This is different in the case of clays, which can be recommended for use both externally, sometimes on very extensive areas of skin [7], and in some cases internally [22,23]. This may increase the body’s exposure to Hg. The process of absorption through the skin is influenced, in addition to the frequency of use, by the amount of product and the skin’s hydration [71]. The use of aqueous cosmetic clay solutions, especially on larger areas of the body, promotes the bodily absorption of both beneficial ingredients and pollutants.

In the literature on the safety assessment of naturally sourced clays used in cosmetics, it was determined that such products contain heavy metals in amounts not exceeding 0.5 mg/kg antimony, 17 mg/kg arsenic, 0.5 mg/kg cadmium, 7 mg/kg cobalt, 0.5 mg/kg tin, 0.05 mg/kg mercury, 20 mg/kg nickel, and 20 mg/kg lead [68]. Additionally, in vivo tests were conducted using human skin models, and the metal penetration of vanadium, lead, arsenic, barium, nickel, chromium, and aluminum was determined. No heavy metals were detected in the diffusion or storage liquids, while that in clay pastes did not penetrate cutaneous tissue [68]. In the above studies [68], no information on Hg is provided, and as suggested by the results of Sin and Tsang (2003) [63], Hg contained in cosmetics may result in an increased level of this metal in the body. Studies indicate that a potential health risk associated with exposure to heavy metals during the use of cosmetics cannot be ruled out [72]. According to Gomes et al. (2021) [66], topically applied clays may cause exposure to toxicity due to persistent skin adsorption of potentially toxic elements and compounds. This is of particular importance and needs to be emphasized, because the quantity, frequency and variety of cosmetics used by consumers is gradually increasing [50]. Continuous monitoring and research on the presence and effect of metals in cosmetics is essential for the protection of public health [68]. Such studies should be detailed and comprehensive, covering as many products as possible, because individual types of clay differ statistically significantly in terms of Hg content, as shown in this study. Mattioli et al. (2016) [51] determined that in terms of the metal content (zinc, lead, arsenic, and barium), white clay is the most dangerous to human health. Moreover, we found that the average Hg content in white clay (AM = 52.8 µg/kg) was one of the highest values, being only slightly lower than that in green clay (AM = 53.26 µg/kg). In addition, both white and green clay samples were characterized by a large feature variability. The maximum Hg content in the tested samples was three times higher and amounted to 158.41 µg/kg. It should also be noted that statistically significant differences in Hg content were also found for the same type of clay from different manufacturers. This indicates that cosmetic clays are a highly diversified product in terms of Hg content. In the case of this product, random testing may prove insufficient, and further studies should therefore be continued.

Consumers should be aware of the potential health risk associated with the use of cosmetic products [73]. Moreover, Chan T. (2011) [74] suggests that due to the toxicity of Hg, its use in products should be prohibited. It should also be noted that the use of cosmetics containing Hg not only affects people who directly use these products, but also has a wider range of effects. Indirectly, it affects other people in the environment [72,74] and acts globally on the ecosystem, as Hg compounds released from cosmetics via their use or production are introduced into the environment, where they can be transformed into organic forms of metal, such as methylmercury, with greater biological availability and toxicity.

## 5. Conclusions

In all tested samples of cosmetic clays, the presence of Hg was found in the range of 1.87–200.8 µg/kg. The variability in Hg content in individual types of clay was large, especially across samples of white and green clay.

Due to the presence of Hg found in all samples of cosmetic clay tested, and the high variability in metal content, it is indicated that cosmetic clays need to be further tested for metal concentration.

None of the Hg contents in the analyzed cosmetic clay samples exceeded the permissible standards for metal content in cosmetics, but it cannot be ruled out that potential risks to the body still remain. Recommendations for the use of cosmetic clays both externally, including on large expanses of skin, and internally may increase the body’s exposure, and long-term use may lead to Hg accumulation and various health problems.

For public health reasons, it is necessary to test clays and other cosmetic products for Hg and other heavy metals. Cosmetics are often forgotten or downplayed as potential sources of exposure, while current legal regulations, often ignored and underestimated, are insufficient to ensure an adequate level of safety.

## Figures and Tables

**Table 1:** Number of samples (N) of tested cosmetic clay types with regard to the manufacturer.

	Manufacturer										
	Yasumi (Kalisz, Poland	Olvita (Marcinowice, Poland	Nacomi (Wilkowice, Poland)	NaturPlanet (Katowice, Poland)	MyBuddy (Rzeszow, Poland)	EcoFlores (Nowy Targ, Poland)	EcoSpa (Warszawa, Poland)	Your Natural Side (Krakow, Poland)	Nature Queen (Poznan, Poland)	Total N	%
Red	3	3	3			3		3	3	18	16
Green	3		3	3	3	3		3	3	21	19
Purple						3	3	3	3	12	11
Blue						3		3	3	9	8
Rose						3		3	3	9	8
White			3			3	3	3	3	15	14
Black			3			3				6	5
Yellow						3	3	3	3	12	11
Rhassoul						3		3		6	5
Orange							3			3	3
Total	6	3	12	3	3	27	12	24	21	111	100

**Table 2:** Statistical analysis of Hg content [µg/kg] in cosmetic clays.

Clay	N	AM ± SD	Me	Min	Max	Quartile		CV [%]
						Q_1_	Q_3_	
White	15	52.80 ± 64.97	5.31	2.31	158.41	3.51	99.39	123
Red	18	19.71 ± 10.20	21.29	5.82	33.63	7.26	28.09	52
Black	6	21.79 ± 20.75	21.88	2.38	40.98	3.02	40.62	95
Purple	12	2.56 ± 0.46	2.54	1.87	3.31	2.24	2.88	18
Blue	9	3.69 ± 0.52	3.52	3.12	4.61	3.32	4.08	14
Orange	3	27.24 ± 2.31	26.52	25.38	29.83	25.38	29.83	9
Rose	9	10.08 ± 1.38	9.56	8.36	12.63	9.37	11.03	14
Rhassoul	6	38.66 ± 9.80	38.72	29.23	48.26	29.67	47.38	25
Green	21	53.26 ± 57.45	32.24	13.69	200.81	29.41	38.64	108
Yellow	12	28.74 ± 1.96	28.03	26.73	32.58	27.14	29.64	7
All	111	28.91 ± 39.34	23.72	1.87	200.81	4.08	32.05	136

N—number of samples, AM—arithmetic mean, SD—standard deviation, Min—minimum, Max—maximum, Q_1_—lower quartile, Q_3_—upper quartile; CV—coefficient of variation.

## Data Availability

The original contributions presented in this study are included in the article. Further inquiries can be directed to the corresponding author.
